# Intrauterine Pressure Catheter in Labor: Associated Microbiology

**DOI:** 10.1155/S1064744993000158

**Published:** 1993

**Authors:** Phillip Pinell, Sebastian Faro, Scott Roberts, Sy Le, Maurizio Maccato, Hunter Hammill

**Affiliations:** 1 Department of Obstetrics and Gynecology Baylor College of Medicine Houston TX USA; 2 Department of Obstetrics and Gynecology University of Texas Southwestern Medical Center Dallas TX USA; 3 Department of Gynecology and Obstetrics University of Kansas School of Medicine Kansas City KS USA; 4 Department of Obstetrics and Gynecology Group Medical Health Associates Tucson AZ USA; 5 Department of Obstetrics and Gynecology Baylor College of Medicine One Baylor Plaza Houston TX 77030 USA

## Abstract

*Objective:* The purpose of this study was to determine if bacterial growth occurred in the amniotic fluid of laboring women. Twenty patients who required an intrauterine pressure catheter (IUPC) during labor were studied. Amniotic fluid samples were aspirated during labor and at the time of delivery.

*Methods:* IUPCs were placed in laboring patients for a variety of reasons. Cervical cultures were taken prior to insertion of an IUPC. After the IUPC was placed, amniotic fluid cultures were taken both at the time of placement and 30 minutes prior to delivery. These cultures were sent for aerobic, anaerobic, *Mycoplasma*, and *Ureaplasma* cultures.

*Results:* The increase in bacterial concentration from the initial sample to the final sample was statistically significant (*P* < 0.01) for both aerobes and anaerobes. Amniotic fluid samples demonstrated a median of 0 bacterial species per patient on initial collection and 2 bacterial species per patient in final collection. The mean count of cfu for erobes in the initial amniotic samples was 3.5 × 10^4^, compared to that of the second samples, which was 1.4 × 10^5^. The mean count of cfu for anaerobes in the initial amniotic fluid samples,.was 4.1 × 10^2^, compared to that of the second samples, which was 8.0 × 10^3^. Only 3 of 20 patients developed chorioamnionitis, with only 1 patient having an increased number ofbacterial species significantly higher than the median. Although 80% of patients had a colony count ≥ 10^2^ cfu/cc, only 19% of this group developed chorioamnionitis.

*Conclusions:* The number of bacterial species and colony counts increased significantly during labor, but this factor alone was not enough to cause chorioamnionitis in a significant number of patients.


Infectious Diseases in Obstetrics and Gynecology 1:60–64 (1993)
© 1993 Wiley-Liss, Inc.

### Intrauterine Pressure Catheter in Labor: Associated Microbiology

**Phillip Pinell, Sebastian Faro, Scott Roberts, Sy Le, Maurizio Maccato, and Hunter Hammill**

*Department of Obstetrics and Gynecology, Baylor College of Medicine, Houston (P.P., M.M., H.H.), Department of Obstetrics and Gynecology, University of Texas Southwestern Medical Center, Dallas (S.R.), TX, Department of Gynecology and Obstetrics, University of Kansas School of Medicine, Kansas City (S.F.), KS, and Department of Obstetrics and Gynecology, Group Medical Health Associates, Tucson (S.L.), AZ*

### Abstract

***Objective:*** **The purpose of this study was to determine if bacterial growth occurred in the amniotic fluid of laboring women. Twenty patients who required an intrauterine pressure catheter (IUPC) during labor were studied. Amniotic fluid samples were aspirated during labor and at the time of delivery.**

***Methods:*** **IUPCs were placed in laboring patients for a variety of reasons. Cervical cultures were taken prior to insertion of an IUPC. After the IUPC was placed, amniotic fluid cultures were taken both at the time of placement and 30 minutes prior to delivery. These cultures were sent for aerobic, anaerobic, *Mycoplasma*, and *Ureaplasma* cultures.**

***Results:*** **The increase in bacterial concentration from the initial sample to the final sample was statistically significant ($P < 0.01$) for both aerobes and anaerobes. Amniotic fluid samples demonstrated a median of 0 bacterial species per patient on initial collection and 2 bacterial species per patient in final collection. The mean count of cfu for aerobes in the initial amniotic samples was $3.5 \times 10^4$, compared to that of the second samples, which was $1.4 \times 10^5$. The mean count of cfu for anaerobes in the initial amniotic fluid samples was $4.1 \times 10^2$, compared to that of the second samples, which was $8.0 \times 10^3$. Only 3 of 20 patients developed chorioamnionitis, with only 1 patient having an increased number of bacterial species significantly higher than the median. Although 80% of patients had a colony count $\geq 10^2$ cfu/cc, only 19% of this group developed chorioamnionitis.**

***Conclusions:*** **The number of bacterial species and colony counts increased significantly during labor, but this factor alone was not enough to cause chorioamnionitis in a significant number of patients.** © 1993 Wiley-Liss, Inc.

Key words
Intrauterine pressure catheter, bacterial colonization, amniotic fluid, chorioamnionitis, endomyometritis

Intrauterine pressure catheters (IUPCs) are frequently employed to assess labor by monitoring the intensity and frequency of uterine contractions. Recently, the IUPC has been utilized to perform amnioinfusion in situations where the amniotic fluid is significantly decreased to prevent cord compression during uterine contractions.[1] The IUPC may also provide a potential route for endogenous bacteria of the lower genital tract to gain access to the uterine cavity.[2,3] Miller et al.[4] found no differ-

**Address correspondence/reprint requests to Dr. Phillip Pinell, Department of Obstetrics and Gynecology, Baylor College of Medicine, One Baylor Plaza, Houston, TX 77030.**

Received October 2, 1992

Accepted July 6, 1993

Clinical Study

**TABLE 1. Demographics of patient population (N = 20)**

| |
|---|
| Race |
| Black = 3 |
| Hispanic = 13 |
| White = 3 |
| Pakistani = 1 |
| Age |
| Mean = 26.5 years |
| Mode = 24 years |
| Range = 14–40 years |
| Gravidity |
| Mean = 3.2 |
| Mode = 2 |
| Range = 1–9 |
| Parity |
| Mean = 1.2 |
| Mode = 0 |
| Range = 0–6 |
| Vaginal exams |
| Mean = 6.5 |
| Mode = 6 |
| Range = 3–10 |
| Delivery |
| Vaginal (spontaneous) = 12 |
| Vaginal (forceps) = 4 |
| Cesarean section = 4 |
| No. of hours of rupture of membranes |
| Mean = 11 hours 38 minutes |
| Mode = 6.5 hours |
| Range = 2 hours 10 minutes–33 hours 29 minutes |

ences among culture results of amniotic fluid samples obtained by aspiration through an IUPC, amniocentesis, and collection at the time of cesarean section. However, being a qualitative bacteriologic study, it did not reveal the dynamic relationship that may occur between the bacteria and amniotic fluid with respect to time.

The relationship between bacterial colonization of the intrauterine environment and bacterial growth during labor in nulliparous women has been previously described.[5] This study was designed to identify and quantitate the bacterial flora of the intrauterine cavity during labor in patients requiring an IUPC and to examine the effect of time on bacterial colonization.

### MATERIALS AND METHODS

This study was conducted on Baylor College of Medicine's Obstetrics Service at Ben Taub General Hospital, a county hospital that serves the indigent and lower socioeconomic population of Harris County, TX. IUPCs were placed in laboring patients for one of the following reasons: dysfunctional labor (50% of patients), trial of labor with history of cesarean section (25%), amnioinfusion on the basis of frequent occurrence of variable decelerations of the fetal heart rate (15%), or the inability to adequately monitor uterine contractions (10%).

Prior to placement of the IUPC, a cervical specimen was collected with a sterile cotton-tipped applicator and placed in anaerobic brain-heart infusion broth transport media. After the IUPC was in place, amniotic fluid was aspirated for the culture of bacteria. The initial 5 cc of amniotic fluid aspirated was discarded. Then, an additional 3 cc was aspirated and placed in anaerobic brain-heart infusion broth transport media. A second specimen was obtained 30 minutes prior to delivery. All specimens were stored at 4°C immediately after collection and processed within 24 hours. Remel blood agar, chocolate agar, and McConkey's medium were inoculated for the isolation of aerobic bacteria. The following media were inoculated for the isolation of anaerobic bacteria: CDC blood, KVKDK, and PEACDC agar. A-7 medium was inoculated in an attempt to isolate *Mycoplasma* and *Ureaplasma*. Qualitative and quantitative bacteriology were performed on all specimens except *Mycoplasma* and *Ureaplasma* as previously described.[6,7]

The number of vaginal examinations were recorded, commencing at the time of IUPC placement until the time of the second amniotic fluid collection.

For the purposes of this study, chorioamnionitis was defined as an infection of the chorioamniotic membranes and the amniotic cavity clinically manifested by maternal fever ≥38°C, uterine tenderness, and/or foul-smelling amniotic fluid. Postpartum endomyometritis was considered to be an infection of the endometrium or decidua with extension to the myometrium with clinical findings of 2 readings of maternal temperature elevation ≥38°C after the first 24-hour postpartum period, uterine tenderness, and leukocytosis [white blood cell counts (WBCs) >15,000].

### Statistics

The number of bacterial isolates was expressed as means ± standard deviation, and the median number of isolates for each group, aerobes and anaerobes, was determined. The Student's t-test was used

**TABLE 2. Cervical cultures at time of IUPC insertion**

| Aerobes | | | Anaerobes | | |
|---|---|---|---|---|---|
| Species | No. positive cultures | % | Species | No. positive cultures | % |
| *Staphylococcus* not *aureus* | 14 | 23.7 | *Peptostreptococcus anaerobius* | 1 | 7.7 |
| *Diphtheroids* | 14 | 23.7 | *S. morbillorum* | 3 | 23 |
| *Lactobacillus* | 6 | 10.1 | *P. magnus* | 1 | 7.7 |
| *S. aureus* | 2 | 3.4 | *Propionibacterium* | 1 | 7.7 |
| *S. agalactiae* | 3 | 5.1 | *Clostridium* sp. | 1 | 7.7 |
| *Enterococcus faecalis* | 4 | 6.8 | *Bacteroides bivius* | 1 | 7.7 |
| *Gardnerella vaginalis* | 13 | 22.1 | *B. intermedius* | 1 | 7.7 |
| *Pseudomonas aeruginosae* | 1 | 1.7 | *B. oralis* | 2 | 15.4 |
| *Klebsiella pneumoniae* | 1 | 1.7 | *B. melaninogenicus* | 1 | 7.7 |
| *Acinetobacter* | 1 | 1.7 | *Fusobacterium* | 1 | 7.7 |
| Totals | 59 | | | 13 | |

to compare mean numbers of bacterial isolates and concentrations between the initial and second samples. A two-tailed test was employed with $P < 0.05$ considered significant. The median numbers of isolates were compared between the initial and second samples using the Wilcoxon rank-sum analysis.

### RESULTS

The demographic data of the 20 patients in this study are presented in Table 1. Twelve patients (60%) had a spontaneous vaginal delivery, 4 (20%) had forceps vaginal delivery, and 4 (20%) required cesarean section. Two of the patients who were delivered by cesarean section and one who was delivered by low forceps developed chorioamnionitis (3/20 or 15%). All 3 of these patients had oral body temperatures ≥38°C, elevated WBCs, and uterine fundal tenderness. The WBCs on admission for patient nos. 5, 10, and 14 were 9,200, 11,300, and 9,700, respectively. The WBCs at the time chorioamnionitis was diagnosed were 32,300, 17,300, and 12,000, respectively. No patient developed postpartum endomyometritis.

The spectrum of bacterial species from the cervical cultures at the time of IUPC insertion is shown in Table 2. The bacterial flora of the amniotic fluid collected at the beginning and near the time of delivery are listed in Table 3. Quantitative results from amniotic fluid samples at the two collecting times are summarized in Table 4.

*Ureaplasma* was isolated from the cervix of 80% (16/20) of the patients. Forty percent (8/20) of the

**TABLE 3. Amniotic fluid cultures**

| No. of positive cultures | | | |
|---|---|---|---|
| At time of IUPC placement | No. | Within 30 minutes of delivery | No. |
| Aerobes | | | |
| *Lactobacillus* | 4 | *Lactobacillus* | 7 |
| *Diphtheroids* | 3 | *Diphtheroids* | 6 |
| *Staphylococcus* not *aureus* | 3 | *Staphylococcus* not *aureus* | 4 |
| *Enterococcus faecalis* | 1 | *S. aureus* | 1 |
| *S. agalactiae* | 1 | *E. faecalis* | 1 |
| *Klebsiella pneumoniae* | 1 | *K. pneumoniae* | 1 |
| *Gardnerella vaginalis* | 2 | *G. vaginalis* | 6 |
| | | *Escherichia coli* | 2 |
| Totals | 15 | | 28 |
| Anaerobes | | | |
| *S. morbillorum* | 2 | *S. morbillorum* | 2 |
| *Bacteroides intermedius* | 1 | *S. subterminale* | 1 |
| | | *B. oralis* | 1 |
| | | *B. urealyticus* | 1 |
| | | *B. bivius* | 1 |
| | | *Fusobacterium* | 1 |
| | | *Veillonella* sp. | 1 |
| Totals | 3 | | 8 |

amniotic fluid cultures from the initial collection grew *Ureaplasma*, compared to 45% (9/20) of the final fluid samples. *Mycoplasma* was isolated from the cervix of 15% (3/20) of patients. Five percent (1/20) of the amniotic fluid samples at the initial collection and final collection had positive *Mycoplasma* cultures.

The number of different aerobic and anaerobic species per patient from the first and second amni-

**TABLE 4. Quantitative analysis of amniotic fluid bacteria[a]**

| Patient | Initial collection | | | | Final collection | | | |
|---|---|---|---|---|---|---|---|---|
| no. | Aerobes | Anaerobes | Mycoplasma | Ureaplasma | Aerobes | Anaerobes | Mycoplasma | Ureaplasma |
| 1 | $5 \times 10^2$ LB | $2 \times 10^2$ BI | | | $2.4 \times 10^3$ LB | No growth | | |
| 2 | No growth | | | (+) | $3 \times 10^2$ LB<br>$2.2 \times 10^3$ DIP<br>$6 \times 10^2$ SNA<br>$5 \times 10^3$ GV | $1.2 \times 10^4$ SM | | (+) |
| 3 | $10^5$ GV<br>$3.5 \times 10^3$ SNA<br>$2 \times 10^3$ DIP | | (+) | (+) | $10^6$ GV<br>$5 \times 10^2$ SNA<br>$2 \times 10^2$ DIP<br>$5 \times 10^4$ LB | $2 \times 10^2$ V | (+) | (+) |
| 4 | No growth | | | (+) | $1 \times 10^4$ LB<br>$10^5$ GV | | | (+) |
| 5 | No growth | | | | $10^6$ GV | $10^5$ F | | (+) |
| 6 | $4 \times 10^3$ KP | | | | $5 \times 10^4$ KP | | | |
| 7 | $2 \times 10^4$ LB<br>$10^4$ DIP<br>$7 \times 10^3$ SNA | | | | $2.4 \times 10^4$ LB<br>$1 \times 10^4$ SNA | | | |
| 8 | No growth | | | | No growth | | | |
| 9 | $5 \times 10^5$ LB<br>$1.2 \times 10^3$ GBBS | | | | $8 \times 10^3$ LB<br>$5 \times 10^5$ GBBS | | | |
| 10 | $3 \times 10^4$ DIP<br>$6 \times 10^3$ GV<br>$1 \times 10^4$ LB | $7 \times 10^3$ SM | | (+) | $3 \times 10^3$ DIP<br>$1.2 \times 10^4$ GV<br>$1 \times 10^3$ LB | $2 \times 10^3$ SM | | (+) |
| 11 | $4 \times 10^3$ EF | | | (+) | $2 \times 10^3$ EF<br>$3 \times 10^3$ DIP | $1 \times 10^4$ BIV | | (+) |
| 12 | $4 \times 10^3$ SNA | | | | | | | |
| 13 | No growth | | | | No growth | | | |
| 14 | No growth | | | (+) | $8 \times 10^3$ LB<br>$1 \times 10^3$ DIP | | | (+) |
| 15 | No growth | | | (+) | $2 \times 10^3$ SA<br>$1.4 \times 10^4$ DIP<br>$5 \times 10^3$ GV | $9 \times 10^3$ CS | | (+) |
| 16 | No growth | | | | No growth | $2 \times 10^3$ BU | | |
| 17 | No growth | $1 \times 10^3$ SM | | (+) | $2 \times 10^3$ SNA | $5 \times 10^3$ BO | | (+) |
| 18 | No growth | | | | $1 \times 10^3$ GBBS | | | |
| 19 | No growth | | | | No growth | | | |
| 20 | No growth | | | | $1 \times 10^3$ EC | | | |

[a]BI = *Bacteroides intermedius;* BIV = *B. bivius;* BO = *B. oralis;* BU = *B. urealyticus;* CS = *Clostridium* sp.; DIP = *Diphtheroids;* EC = *Enterococcus;* EF = *E. faecalis;* F = *Fusobacterium;* GBBS = group B *beta-strep;* GV = *Gardnerella vaginalis;* KP = *Klebsiella pneumoniae;* LB = *Lactobacillus;* SA = *Staphylococcus aureus;* SM = *S. morbillorum;* SNA = *Staphylococcus* not *aureus;* V = *Veillonella* sp.

otic fluid samples varied from 0 to 4 species (Table 4). The mean number of bacterial species in the initial fluid samples was 0.9 and the median was 0. The mean number of bacterial species in the final fluid samples was 1.8 and the median was 2. The mean count of cfu for aerobes in the initial amniotic fluid samples was $3.5 \times 10^4$, compared to that of the second sample, which was $1.4 \times 10^5$. This increase in bacterial count was statistically significant ($P < 0.01$). Likewise, the mean count of anaerobic cfu from the first collection was $4.1 \times 10^2$, compared to that of the second sample, which was $8.0 \times 10^3$. This increase was also statistically significant ($P < 0.01$).

### DISCUSSION

IUPCs are used in laboring patients for monitoring purposes and for performing amnioinfusions. Because they are placed transvaginally, the possibility exists of introducing vaginal flora into the amniotic cavity. The lower genital tract, especially the vagina, represents a unique microsphere of microbiological life.[8] Surveying the microbiology of the genital tract during labor is important to determine the dynamics of the microbiological ecology of amniotic fluid, e.g., which organisms become dominant and their relationship to the development of chorioamnionitis and endomyometritis. In patients with acute chorioamnionitis, a polymicrobial contamination of the amniotic cavity occurs following rupture of membranes and labor. Gilstrap and Cunningham[9] reported a mean per patient of 2.5 microorganisms, 72% of which were gram-positive cocci. In this study, a mean of 0.9 bacterial species was noted, and final samples demonstrated a mean of 1.8 bacterial species per patient. Of the 3 of 20 patients who developed chorioamnionitis in this study, only 1 patient had bacterial species numbers significantly higher than the means noted.

Inoculum size is a significant factor in the development of chorioamnionitis and postpartum endomyometritis. Both Miller et al.[4] and Gibbs et al.[10] demonstrated that intraamniotic infection occurred with greater than $10^2$ cfu. While 80% of the patients in our study had colony counts $\geq 10^2$ cfu, only 19% of this group developed chorioamnionitis. Thus, colonization alone appears not to be the only significant factor. According to Larsen et al.,[11,12] the amniotic fluid contains an active bacterial inhibitor. It was noted in our study that growth occurred despite this amniotic fluid inhibitor. Although bacterial growth occurs, clinical evidence of an infection does not necessarily ensue. Clinical infection may depend on a number of issues, such as pathogenicity (virulence) and tissue invasion. Concentration may also play a role in this process.

In conclusion, our study demonstrated that both the number of bacterial species and the quantitative cfu increase significantly during labor. This factor alone was not enough to result in chorioamnionitis or postpartum endomyometritis in all patients.

### REFERENCES

1. Miyazola FS, Taylor NA: Saline amnioinfusion for relief of variable or prolonged decelerations. Am J Obstet Gynecol 146:670–685, 1983.
2. Levison ME, Corman LC, Carrington ER, Kaye D: Quantitative microflora of the vagina. Am J Obstet Gynecol 127:80–85, 1977.
3. Bartlett, JG, Moon NE, Goldstein PR, Goren B, Onderdonk AB, Polk BF: Cervical and vaginal flora: Ecologic niches in the lower female genital tract. Am J Obstet Gynecol 130:658–661, 1978.
4. Miller JM Jr, Hill GB, Welt SI, Pupkin MJ: Bacterial colonization of amniotic fluid in the presence of ruptured membranes. Am J Obstet Gynecol 137:451–458, 1980.
5. Silver RK, Gibbs RS, Castillo M: Effect of amniotic fluid bacteria on the course of labor in nulliparous women at term. Obstet Gynecol 68:587–592, 1986.
6. Phillips LE, Faro S, Martens MG, et al.: Postcesarean microbiology of high risk patients treated for endometritis. Curr Ther Res Clin Exp 42:1157–1165, 1987.
7. Faro S, Phillips LE, Baker JL, Goodrich KH, Turner RM, Riddle GD: Comparative efficacy and safety of mezlocillin versus cefoxitin versus clindamycin plus gentamicin in the treatment of patients with postpartum endometritis. Obstet Gynecol 69:760–766, 1987.
8. Gilstrap LC, Cunningham FG: Microflora of the genital tract. In Gilstrap LC, Faro S (eds): Infections in Pregnancy. New York: Wiley-Liss, pp 1–5, 1990.
9. Gilstrap LC, Cunningham FG: Single drug regimen proves effective in postcesarean infection. Contemp Ob/Gyn 14:68–75, 1979.
10. Gibbs RS, Blanco JC, St Clair PJ, Castaneda YS: Quantitative bacteriology of amniotic fluid from patients with clinical intraamniotic infection at term. J Infect Dis 145: 1–8, 1982.
11. Larsen B, Snyder IS, Galask RP: Bacterial growth inhibition by amniotic fluid. I. In vitro evidence for bacterial growth inhibiting activity. Am J Obstet Gynecol 119: 492, 1974.
12. Larsen B, Snyder IS, Galask RP: Bacterial growth inhibition by amniotic fluid. II. Reversal of amniotic fluid bacterial growth inhibition by addition of a chemically defined medium. Am J Obstet Gynecol 119:497, 1974.
